# Influence of High-Intensity Interval Training on Neuroplasticity Markers in Post-Stroke Patients: Systematic Review

**DOI:** 10.3390/jcm13071985

**Published:** 2024-03-29

**Authors:** Gines Montero-Almagro, Carlos Bernal-Utrera, Noelia Geribaldi-Doldán, Pedro Nunez-Abades, Carmen Castro, Cleofas Rodriguez-Blanco

**Affiliations:** 1 Physiotherapy Department, Faculty of Nursing, Physiotherapy and Podiatry, University of Seville, 41013 Seville, Spain; gmontalgro@gmail.com (G.M.-A.); cleofas@us.es (C.R.-B.); 2 Institute for Biomedical Research and Innovation of Cadiz (INiBICA), 11009 Cadiz, Spain; pnunez@us.es (P.N.-A.); carmen.castro@gm.uca.es (C.C.); 3 Department of Human Anatomy and Embryology, Faculty of Medicine, University of Cadiz, 11002 Cadiz, Spain; noelia.geribaldi@gm.uca.es; 4 Department of Physiology, Faculty of Pharmacy, University of Seville, 41013 Seville, Spain; 5 Department of Biomedicine, Biotechnology and Public Health, Area of Physiology, Faculty of Medicine, University of Cadiz, 11002 Cadiz, Spain

**Keywords:** exercise, stroke, high-intensity interval training, physiotherapy, neuroplasticity, exerkines

## Abstract

**Background:** Exercise has shown beneficial effects on neuronal neuroplasticity; therefore, we want to analyze the influence of high-intensity interval training (HIIT) on neuroplasticity markers in post-stroke patients. **Methods:** A systematic review of RCTs including studies with stroke participants was conducted using the following databases (PubMed, LILACS, ProQuest, PEDro, Web of Science). Searches lasted till (20/11/2023). Studies that used a HIIT protocol as the main treatment or as a coadjutant treatment whose outcomes were neural plasticity markers were used and compared with other exercise protocols, controls or other kinds of treatment. Studies that included other neurological illnesses, comorbidities that interfere with stroke or patients unable to complete a HIIT protocol were excluded. HIIT protocol, methods to assess intensity, neuroplasticity markers (plasmatic and neurophysiological) and other types of assessments such as cognitive scales were extracted to make a narrative synthesis. Jadad and PEDro scales were used to assess bias. **Results:** Eight articles were included, one included lacunar stroke (less than 3 weeks) and the rest had chronic stroke. The results found here indicate that HIIT facilitates neuronal recovery in response to an ischemic injury. This type of training increases the plasma concentrations of lactate, BDNF and VEGF, which are neurotrophic and growth factors involved in neuroplasticity. HIIT also positively regulates other neurophysiological measurements that are directly associated with a better outcome in motor learning tasks. **Conclusions:** We conclude that HIIT improves post-stroke recovery by increasing neuroplasticity markers. However, a limited number of studies have been found indicating that future studies are needed that assess this effect and include the analysis of the number of intervals and their duration in order to maximize this effect.

## 1. Introduction

According to the American Heart Association and American Stroke Association, stroke includes any condition in which there is demonstrated evidence of permanent brain, spinal cord or retinal cell death due to a vascular cause [1]. In stroke, after the loss of oxygen and glucose, energy deprivation and other cascade reactions lead to the dilation of vessels, neuroinflammation and finally necrosis [2]. This inflammatory process starts a few minutes after the episode and can last weeks or months leading to the late death of neurons [2]. While this process is happening, surrounding the infarcted area (even the core of the stroke), there is a zone called penumbra where ischemia is reversible but depends on residual blood flow and time without blood. With time, necrosis extends to the penumbra, taking its place [3,4].

One of the main strengths of the nervous system to recover from this condition is neuroplasticity, which consists of the use of several cell groups to reorganize and create new neural networks modulated by the stimuli received [2,5]. Different forms of neuroplasticity can be found which vary from neurogenesis to the adjustment of synapses, remodulating the structure and function of neural networks [5]. Recent reports highlight the role of signaling molecules released in response to physical exercise that is described under the name of “exerkines”. Exerkines are signaling molecules that belong to different categories such as cytokines (IL-6, IL-8, musclin), growth factors (VEGF, TGFβ2), neurotrophins (BDNF, NT-3) hormones (Irisin) or metabolites (lactate, β-hydroxybutyrate (DBHB)) [6]. Among these exerkines, we would like to highlight the role of the brain-derived neurotrophic factor (BDNF), the vascular endothelial growth factor (VEGF) and lactate, which have been reported to induce the activation of the neuroplasticity mechanism and indicate the level of neuroplastic activity [7,8].

It has been observed that plasma levels of BDNF decrease in stroke patients [9,10,11], as it happens in other pathologies like atherosclerosis, diabetes mellitus and metabolic syndrome [11]. Moreover, these low BDNF levels are associated with a higher risk of stroke and transient ischemic attack and low recovery rates [11]. It has been observed that BDNF plays an important role in dendrite growth and neurotransmitter regulation enhancing neuroplasticity [7,9,10]. VEGF is another growth factor that is autoregulated after an aggression to the CNS. This growth factor promotes angiogenesis (especially in the case of hypoxia) [12] and increases the permeability of the brain–blood barrier [8]. VEGF-mediated angiogenesis occurs 4 to 7 days at the edge of the ischemic core, increasing blood flow and allowing nutrients to arrive at the ischemic area [13]. Moreover, these new blood vessels seem to promote new axons and guide their growth by laminin/β1-integrin signalin [14] with the earliest axon appearance after 14 days and cortical circuits after 3 weeks [13]. Lastly, lactate was considered a waste product of anaerobic metabolism for a long time [7,15]. However, now it seems to play a key role in different processes within the CNS, because lactate can cross the blood–brain barrier through monocarboxylic transporters (MCTs) 1–4, mainly the MCT1 [7,15]. In addition, lactate is of vital necessity for neuronal survival and function. Thus, in astrocyte–neuron cocultures without lactate or its transporters, neuronal cell death can be observed [15]. Particularly, an astrocyte–neuron lactate shuttle has been proposed to highlight the energetic role of lactate in the CNS; thus, glutamate intake into neurons and astrocytes is regulated by a sodium transporter that consumes ATP, stimulating glycolysis and generating lactate, which is transported by MCT to neurons and astrocytes where it is transformed into pyruvate and used to synthesize acetyl-CoA [15].

In this context, studies show that voluntary physical exercise increases synaptogenesis and neurogenesis in the cerebral cortex and hippocampus [15,16]. Such beneficial effects are exerted by influencing the neural biomarkers mentioned above. Particularly, it has been observed an increase in BDNF in adults subjected to high-intensity interval training (HIIT) when compared to others that faced moderate-intensity continuous training (MICT) [17]. These findings suggest that HIIT could be an interesting therapy in a multidisciplinary intervention since it may release signaling molecules that stimulate neuroplasticity. However, little is known about the beneficiary effects that HIIT may exert in the recovery of stroke patients. Although it seems reasonable to hypothesize that HIIT-induced BDNF release may be of help in the recovery of stroke patients, the alterations caused by ischemic events may modify the physiological response of the affected area not being comparable to that observed in healthy individuals. Thus, the stroke injury-induced alterations may modify not only the release of neurotrophic factors involved in neuroplasticity but also the pathways used by these factors to induce a response to exercise and their functions.

Therefore, based on the abovementioned findings, it seems reasonable to hypothesize here that HIIT increases neuroplasticity markers in stroke patients, thus being the main objective of this systematic review—to assess whether HIIT influences neuroplasticity markers in patients affected by stroke.

## 2. Materials and Methods

This systematic review was registered in PROSPERO with registration number CRD42022318598 and was made following the recommendations of PRISMA [18].

### 2.1. Search Strategy

Searches were conducted on the following databases: PubMed, LILACS, ProQuest, PEDro and Web of Science (WOS), with the addition of manual cross-referencing from the articles searched. The project started on (18 March 2022) and searches lasted till (25 June 2022); an update of the search was made and the search was expanded till 20 November 2023.

Search filters were used when available. The following filters were used: time (2017–2023), RCT, article, human and scientific journal. Duplicates were eliminated by matching titles and authors. The restriction on studies before 2017 was applied due to the fact that before 2017 BDNF, HIIT and stroke were not studied in depth; instead, there are feasibility studies that lead to the studies on this systematic review. See Appendix B for further information.

The inclusion and exclusion criteria were defined using the PICOS question acronym, (P: Population, I: Intervention, C: Comparison, O: Outcomes, S: Study Design). The inclusion criteria were randomized clinical trial studies whose participants were 18 years old or older with stroke diagnosed by a physician, which uses a HIIT protocol as the main treatment or as a coadjutant treatment whose outcomes were neural plasticity markers, regardless of them being neurophysiological or plasma markers and comparing them with other exercise protocols, controls or other kinds of treatment. The exclusion criteria were studies that included other neurological illnesses that are not a stroke, patients with comorbidities that interfere with the stroke condition or patients whose actual status renders them unable to complete a HIIT protocol.

### 2.2. Article Search and Selection Process

Two researchers (GM and NG) searched titles and abstracts according to the PICO question. MESH terms were used, adapting the search strategy to the different database requirements. For those studies that meet the requirements, full text was obtained. If there were any doubts about a study meeting the requirements, full text was consulted. If needed, the original author of the text would be contacted. Full text was applied to the same eligibility criteria.

The articles were included if both reviewers agreed. In case of disagreement, they would meet and discuss to reach an agreement. If after that, they still failed to reach an agreement, a third independent researcher (CB) was consulted to determine the inclusion or exclusion of the text applying eligibility criteria. The reviewers were not blinded to the titles of the magazines nor the author’s names of the texts.

### 2.3. Data Processing

Data were extracted by an independent researcher (GM). Extracted data were revised by a second researcher (CB). The following data were extracted: name of the authors, year of publishing, type of HIIT protocol, the method to assess the intensity of the exercise, neuroplasticity markers, time passed since the cerebrovascular episode and other types of assessments such as cognitive scales or quality of life scales.

A narrative synthesis of the outcomes was made, broadly categorized as follows:

- HIIT protocols
  ○ Frequency of sessions
  ○ Number of intervals
  ○ Intensity
- Plasmatic neuroplasticity markers (such as BDNF, VEGF or lactate among others)
- Neurophysiological neuroplasticity markers (such as corticospinal excitability, cortical silent period or motor-evoked potentials)
- Other assessments (cognitive or quality of life scales)
- Demographic data (number of participants, gender and type of stroke)

Two of the studies [19,20] used the same population.

### 2.4. Risk of Bias

The risk of bias and quality of studies were assessed using the PEDro scale for RCTs which is an 11-item scale to assess internal validity (items 2–9), if studies have sufficient statistical information (item 10) and to interpret results (item 11). The first item assesses external validity but it does not account for the total score [21]. The Jadad scale is a 5-point scale to assess the quality of RCTs and it consists of three items: the first item evaluates randomization, the second one blinding and the third one the losses on follow up [22].

Two independent reviewers (GM and NG) applied individually the different scales to the selected studies. In case of a difference between scores, both reviewers would discuss in order to reach an agreement. If they could not reach a consensus, a third independent reviewer (CB) would participate in the discussion to reach an agreement about the score.

Cohen’s k for the PEDro and Jadad scales were evaluated.

## 3. Results

**Figure 1:**
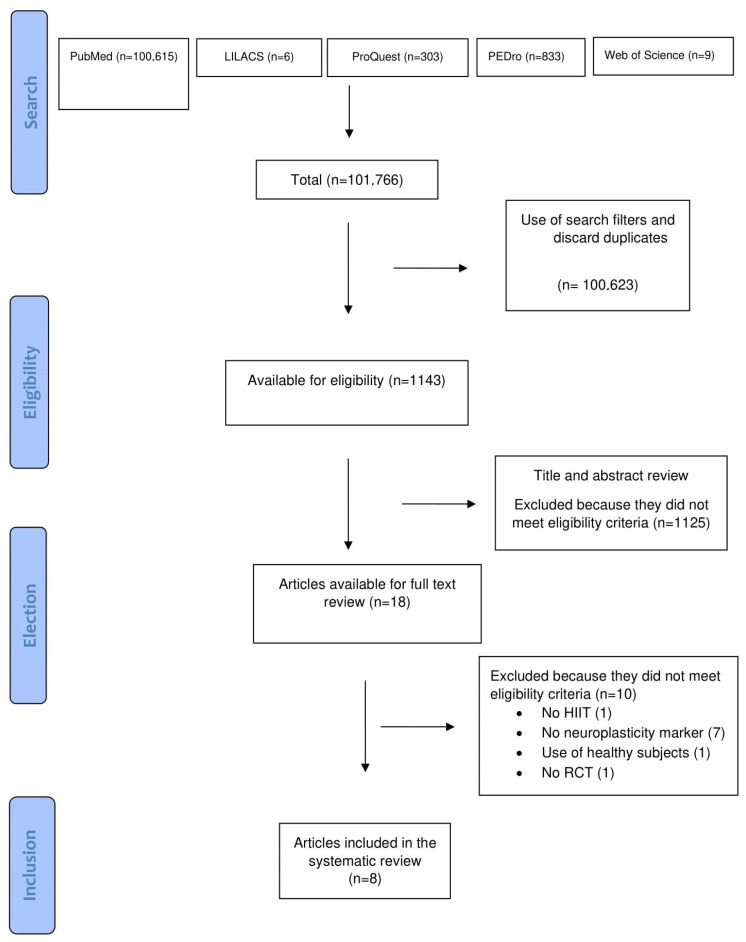
Flow of studies during the review. Articles may be excluded for failing to meet more than one inclusion criteria.

In total, 101,766 articles were retrieved from the searches, after applying filters and eliminating the repeated articles. A total of 1143 results were obtained. Once the initial evaluation was completed, a total amount of 174 studies remained. Once the full-text review was completed, 10 studies were discarded because they did not meet the inclusion criteria. In the end, a total of eight studies were included in the review. The exclusion of studies was made for different reasons: no neuroplasticity markers studied (7), review article (1), use of healthy subjects (1) and no HIIT intervention used (1). See Figure 1.

Eight studies were included in this review [19,20,23,24,25,26,27,28], and five of them compared HIIT with MICT [19,20,25,26,27]. Moreover, HIIT was compared with usual care [24], the improvements in motor tasks to assess skill retention [23,28] and upper limb performance [26,28].

### 3.1. Study Characteristics

The majority of the population of the studies (91%) had chronic stroke (>6 months), which makes 87.5% of the studies of this systematic review, with the types of stroke being ischemic and hemorrhagic [19,20,23,25,26,27,28]. Only one study analyzed lacunar strokes less than 3 weeks after the episode, with the addition of home-based treatment [24]. The total amount of participants was 211 with a male/female ratio of 72% males (151)/28% females (60), the distribution of stroke types were 42% (87) for ischaemic, 19% (41) hemorrhagic, 30% (63) lacunar, 9% (20) ischaemic/hemorrhagic (not specified). The majority of the studies used the graded exercise test (GTX) to assess the base cardiovascular conditions of the patients. Although each study used a different device, recumbent stepper [23,26], treadmill [19,20,27] and bicycle ergometer [25]. Only one study used a different test, the graded cycling test with Talk Test [24], and another one used the incremental cycle ergometer test [28]. Only two studies used HIIT protocols with more than one machine, and these machines were a treadmill and a stepper [19,20]. The variety of devices used to perform the HIIT protocol was as follows: a bicycle ergometer [24,25,28], a stepper [19,20,23,26] and lastly a treadmill [19,20,27]. Another study used, in addition to a treadmill, overground walking [27]. See Table 1.

### 3.2. Frequency of Sessions

The majority of the studies used several sessions, varying the number of sessions between 3 [19,20], 30 [28], 36 [25,27] and 60 [24]. Two studies used a single bout of HIIT [23,26]. The frequency of sessions also varied, being 2–3 times per week [25], 3 times a week [27,28], 5 times a week [24] and once a week [19,20].

### 3.3. HIIT Intervals

The most common length of the intervals was 3 min [23,24,25], with the main difference in the number of intervals, which varied between 3 [23,24] and 2 [25], except in the study of Valkenborghs et al. (2019) whose number of the interval was four with a duration of 4 min [28]. Meanwhile, the length of the resting periods varied between 3 [25,28] and 2 min [23,24,26].

On the other hand, in the studies from Boyne et al. (2019, 2020, 2023), 20 min sessions were made, with HIIT intervals of 30 s, using passive recovery resting periods (device stopped) of 60 s and then 30 s after the first 5 min of exercise [19,20,27]. Abraha et al. (2018) also made a 20 min session with 2 min intervals (exercise and recovery) [26]. The majority of the studies had a warm-up period, with the length of these 2 min [23], 3 min [19,20,25,27] and 5 min [26]. In addition, there was an active cooldown period at the end of the training that lasted 2 [27] and 3 min [19,20,25].

### 3.4. Intensity Measures

Nepveu et al. (2017) used the maximum intensity reached in the GTX as a reference, whereby warm up and active recovery is 25% of that intensity, meanwhile, the high-intensity intervals used 100% of that intensity reached in the GTX [23]. Krawcyk et al. (2019) used the Talk Test (reach an intensity where talking is difficult for the person exercising) to assess the initial intensity and proceed to intensity increment from this point; such test corresponds to a 14–16 score in the RPE scale and with the 77–93% of maximum heart rate [24].

Boyne et al. (2019, 2020) used 25% of the heart rate reserve (HRR), afterwards, during the high-intensity period, the treadmill increased speed till the patient showed signs of not being able to keep up or gating instability. The treadmill speed was adjusted in each interval according to the patient’s condition. In the stepper, the high-intensity intervals were performed at the maximum possible cadence at 50% of the maximum resistance. The resistance was adjusted at the end of each high-intensity interval, rising or decreasing it according to the patient’s cadence. The revised HIIT stepper protocol changed resistance for heart rate at an average intensity of 70% and a maximum of 85% [19,20].

Hsu et al. (2021) used VO2 peak to establish the intensity of the exercise, with warm-up and cooldown being 30% of that intensity. High-intensity periods used 80% and active recovery periods used 40%. Intensity was increased every two weeks by 10% of the heart rate reserve according to the patient’s tolerance levels [25].

Abraha et al. (2018) also used VO2 peak to establish intensity, warm up consisted of gradually increasing load to 80% of VO2 peak, recovery periods at 40% and maintaining a step cadence of 60 to 80 [26].

Boyne et al. (2023) used the HRR to establish the intensity of the exercise, maintaining a mean intensity above 60% of HRR during the high-intensity periods, with a warm-up and a cooldown of 30–40% of the HRR [27].

Valkenborghs et al. (2019) used the results of the incremental cycle ergometer test as a base for intensity and then used VO2 peak and workload from the incremental cycle ergometer test to increase the intensity of the interval [28].

To assess if patients’ intensity was kept at the desired levels, the authors used several methods: they mainly used RPE (6–20) [19,20,23] and heart rate [19,20,23,28], although other measurements were used like the Talk Test [24], VO2 [19,20,25,26], plasma levels of lactate [20] and HRR [27].

### 3.5. Plasmatic Neuroplasticity Markers

Two reports found statistically significant increases in plasma levels of BDNF after the application of their HIIT protocols when compared to MICT (3.9 [0.1, 7.8]) [19,20,25]. The revised HIIT stepper protocol from the studies of Boyne et al. (2019, 2020) did not show any statistically significant differences with the MICT protocol (2.9 [−1.0, 0.7]) but the original stepper protocol did (4.4 [0.2, 8.5]) [19,20]. Meanwhile, the MICT protocol from the study of Hsu et al. (2021) decreased serum BDNF levels [25]; in addition to this, serum BDNF levels also decreased in the study of Valkenborghs et al. (2019) with a larger decrease in the group with only task-specific training (22.4 (12.6)→17.7 (8.7)) than the group which combined it with HIIT (24.1 (12.9)→20.4 (12.1)) [28].

Two studies assessed plasma lactate after the HIIT intervention, observing greater increases in lactate in the HIIT protocols when compared to the MICT protocol ((4.6 > 2.0) [20,27] and (1.1 [0.4, 1.8]) [27]). Two studies evaluated the VEGF plasma concentrations, finding inconclusive results: one of them found statistically significant increases in the treadmill HIIT protocol (49.2 [8.2, 90.2]) [20] and the other study found no difference between groups (30.3 [22.5;42.7]) [24]. The main differences between studies were the tool of exercise (treadmill vs. stationary bicycle), time of HIIT (20 min vs. 15 in total), type of session (monitored vs. home-based) and lastly the type of stroke (ischemic vs. lacunar). These differences could explain the results regarding concentrations of plasma VEGF because the study of Boyne et al. (2020) had a higher time, and maybe a higher intensity exercise because sessions were monitored and not at home [20].

Other signaling molecules, cytokines, growth factors or metabolites tested in these studies were as follows: insulin-like growth factor type 1 (IGF-1) [20], interleukin 6 (IL-6), tumor necrosis factor (TNF), intercellular adhesion molecule-1 (ICAM-1), vascular cell adhesion molecule-1 (VCAM-1), E-selectin [24], cerebral tissular oxyhemoglobin, deoxyhemoglobin and total concentration of hemoglobin as well [25].

Statistically significant changes were only found in the concentrations of IGF-1 in plasma, which incremented after applying the treadmill HIIT protocols (3.7 [0.8, 6.6]) and both HIIT stepper protocols (4.2 [1.4, 7.1]) [20]. The three plasma hemoglobin measurements (cerebral tissular oxyhemoglobin, deoxyhemoglobin and total concentration of hemoglobin) increased with HIIT protocols but not with MICT [25].

### 3.6. Neurophysiological Neuroplasticity Markers

Three studies evaluated alterations in neurophysiological parameters obtained through transcranial magnetic stimulation [19,23,26]. They found different results between the two studies about the corticospinal silent period, finding a statistically significant decrease in the treadmill HIIT protocol when compared to MICT (−6.5 [−12.6, −0.4]) [19], whereas the other study found no statistical differences at all (0.10 [0.05, 0.14]) [23].

On the contrary, they found that the corticospinal excitability increased in both hemispheres but more on the affected one (2.83 [−1.99, 7.64] vs. 1.63 [0.37, 2.89]), although these increases were not statistically significant. Short-interval intracortical inhibition decreased in the affected hemisphere but only the interhemispheric ratio was statistically significant (1.34 [0.46, 2.23]) [23]. Another study found a significant length of the motor-evoked potential latency in the ipsilesional side after HIIT and joined with the study of Nepveu et al. (2017), finding no statistical differences in resting motor thresholds for both hemispheres with HIIT or MICT [F(1, 7) < 3.73, *p* > 0.10]/[F(1, 11) < 0.24, *p* > 0.63)] [26]. Lastly, another study assessed dendrite growth in cell cultures, which was statistically significantly higher than the growth in the HIIT group when compared to the MICT group (45.2 [35.2–55.2]) [25].

Two studies evaluated the retention motor capacity after a HIIT session on a motor task, one of them showing statistically higher retention and performance in the HIIT group when compared to the control group (unpaired t-test, t(19) = 2.20; *p* = 0.04; effect size d = 0.96) [23], and the other showing twice as much improvement in the HIIT group compared to the group of only task-specific training [28]. Abraha et al. (2018) evaluated upper limb performance with the box and block test finding no difference between protocols [26].

### 3.7. Other Measurements

Several scales were used to assess the cognitive condition: the Montreal Cognitive Assessment (MoCA) [23,24] and the Mini-Mental Test [25], with the MoCA being the most common. Other measures used were the Multidimensional Fatigue Inventory (MF-20), Fatigue Assessment Scale (FAS) [28], World Health Organization Well-Being Index (WHO-5), chronic stress (algometer) [24], Medical Outcomes Study short form (SF-36) [25], International Physical Activity Questionnaire (IPAC) [28], Wolf Motor Function Test (WMFT) [28] and Action Research Arm Test (ARAT) [28]. Two studies used the six-minute walk test to assess walking capacity [27,28].

### 3.8. Adverse Events

Only one study reported adverse events [19]. These authors had to revise their HIIT stepper protocol because of two adverse events that consisted of symptomatic hypotension (grade 2 adverse event) and near syncope during recovery (grade 3 adverse event). Thus, they had to analyze separately the original HIIT stepper and the revised HIIT stepper protocols. Later, these authors determined that the grade 3 adverse event was not caused by the therapy applied, being malnourishment and dehydration were the main causes [19]. Although not an adverse event, the HIIT training in the study of Valkenborghs et al. (2019) had to be adapted to make it feasible for some participants due to their severe or non-ambulatory conditions with them not reaching the 85%Hrmax, instead reaching 72%Hrmax, but with all of them reaching surpassing at least once the target intensity at peak during the program [28].

### 3.9. Risk of Bias

**Figure 2:**
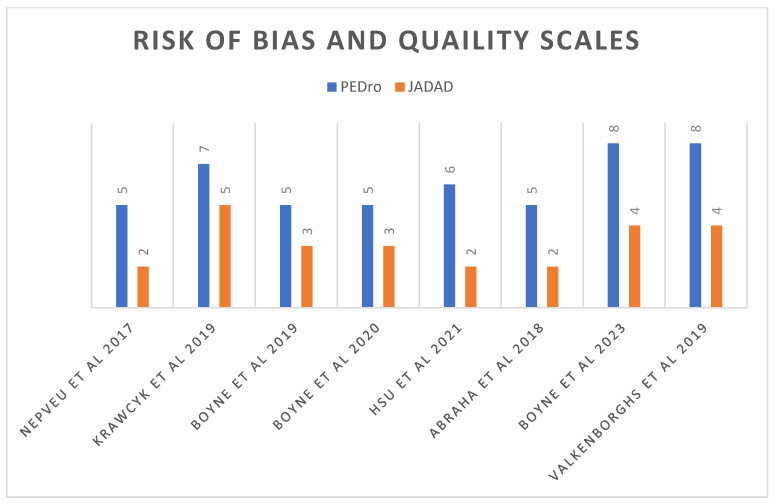
Article scores for quality and risk of bias from the PEDro and Jadad scales [19,20,23,24,25,26,27,28].

The heterogeneity among the studies, apart from the variety of measures, is due to several factors such as different devices for exercise, protocols and a wide range of time passed since the episode. The average score on the Jadad scale was 3.38 and on the PEDro scale 6.13, thus, the level of evidence is acceptable. See Figure 2 and Table 2. Cohen’s k for the PEDro and Jadad scales were 0.49 and 0.47, respectively.

## 4. Discussion

In this work, we analyzed the effect of exercise, particularly HIIT, on the recovery from and ischemic injury. This systematic review highlights that HIIT facilitates neuronal recovery upon an ischemic injury since this training manages to increase the plasma concentrations of neural biomarkers related to neuroplasticity and to positively modify other neurophysiological measurements that are directly associated with a better outcome in motor learning tasks.

Recent reports highlight the role of “exerkines” [6]. A recent review by Sato et al. (2022) defines exerkines as “signaling molecules released in response to acute and/or chronic exercise, which exert their effects through endocrine, paracrine and/or autocrine pathways” and are heavily influenced by exercise modality and timing [29]. There are a few pathways where the highlighted exerkines of this review interact with neuroplasticity regarding the HIIT modality. Physical exercise, especially anaerobic exercise, generates lactate which can cross the blood–brain barrier and increase SIRT1 expression, which then upregulates the expression and release of BDNF [15]. On the other hand, such metabolic stress starts a metabolic cascade that commences with the acetylation of histamines from the IV promoter of BDNF and ends up playing an important role in the modulation of genes related to the metabolism of carbohydrates and fatty acids, among many other functions. Another gene upregulated by exercise is the fibronectin type III domain-containing protein 5 (FNDC5), the precursor of irisin, a protein proposed to be a novel PGC-1α-dependent and exercise-responsive myokine [30]. FNDC5 is also expressed in the brain in response to physical exercise [31] and both compounds PGC-α and irisin favor BDNF secretion on a cerebral level [7,17,32]. Moreover, through hemodynamic stimuli generated by exercise, VEGF is secreted thanks to the effects of transversal stress and tension on the blood vessel walls with the increased blood flow, and heart rate that comes with exercise, especially HIIT [33]. These molecular mechanisms could explain the results obtained in this review. In addition, lactate has an important function in several mechanisms such as being an energetic metabolite for the CNS, indirectly stimulating BDNF production through sirtuins [34] and on the ischemic attacks or hypoxia by activating the expression of the N-myc downstream-regulated gene 3, which stimulates the Ras/Raf/Mitogen-activated protein kinase/extracellular signal-regulated kinase pathway, favoring angiogenesis and cellular growth [15,34].

The analyzed articles confirm that HIIT increases neuroplasticity markers such as BDNF, in agreement with other studies where healthy subjects were subjected to HIIT [35]. Our systematic review focuses mainly on HIIT and expands this line by reassuring these results and linking them to other exerkines that have an influence on neuroplasticity and how they act along the actions of BDNF.

As the main point of this systematic review, we would like to highlight that despite the differences between HIIT protocols of the studies included, all of them managed to increase plasma levels of neuroplasticity markers on a significant level, demonstrating the possible benefit that this type of intervention may exert in patients with stroke as well as its feasibility as a safe and tolerable therapy for stroke patients, given that only one study reported an adverse event which was not attributed to HIIT after investigating it [20].

Regarding the frequency of sessions, some studies lacked long-term evaluation [19,20,23], because it is recommended that protocols last from 4 to 12 weeks to observe the long-term effects despite the initial effects or benefits they could have in a few sessions [36]. Following this, Krawcyk et al. (2023) published a follow-up report of their clinical trial six and twelve months later where the secondary outcomes (among them is VEGF) improved from baseline but with no significant difference [37] as it was stated in the article analyzed in this review [24]. This points towards a need for maintenance in order to keep the beneficial effect of HIIT. Another study with a long follow-up was the one of Valkenborghs et al. (2019) where the effects of HIIT maintained skill retention doubling the one obtained in the other group; however, BDNF was not recorded further than the post-evaluation.

As a recommendation for a HIIT protocol based on the results from this review, we would use maximum heart rate as it is an easily accessible measure, but other more specific measures such as VO2max are recommended. We advise a warm-up of 3 min gradually increasing till the objective intensity. The session will last 20 min as it seems that longer time periods increase the biomarkers, the intervals would last 1 min to maximize the metabolic stress peaks that come with the high-intensity periods with an active recovery period of 1 min, remembering to always take the patient’s condition into account. For the high-intensity periods, we would aim for 75–85% of HRmax and 40% of active recovery periods. Finally, we would have a cooldown of 2 min where we steadily decrease the intensity to basal levels. These indications are a suggestion that can be tested in clinical trials in the future.

Looking into the neural biomarkers analyzed, only two studies analyzed lactate as a main variable [19,20]. This is surprising given the key role that lactate plays in CNS metabolism and in neuroplasticity, the fact that lactate concentrations may be used to assess the intensity of the exercise performed [38], may explain the predetermined assumption of HIIT exercise increasing serum lactate levels [39]. In addition, it could be interesting to take measurements of BDNF, VEGF and lactate shortly after the exercise is finished to assess the duration of the effects in patients with stroke because it is estimated that the increase in BDNF from exercise only lasts for 20 min [40] to 1 h in healthy subjects [41]. From the studies of this review, two of them [25,27] analyzed blood immediately after the exercise, whereas the others from Boyne et al. (2019, 2020) [19,20] did it through the exercise, then 30 and 60 min after the exercise.

Concerning other ways of assessing the effectiveness of HIIT, two studies used a motor learning task to assess the effectiveness of HIIT training [23,28]. Future studies should assess the effects of HIIT on this area in addition to the neuroplasticity markers in order to evaluate the functional recovery and the possible addition of this type of task to the treatment complementing the neuroplasticity provided by HIIT. Following this, another study had a longer session of HIIT which did not yield any results on the upper limb dexterity test [26], as well as was found in the study of Valkenborghs et al. (2019), which had more sessions of HIIT did not yield different results [28]. This may be due to the finer motricity nature of the tasks or the spasticity at the hands that diminishes the effects of exercise therapy. Regarding this, the type of task must be selected with caution because it would be interesting to base the task on the coordination of several body segments stimulating the motor cortex and cerebellum to favor sensorimotor learning [42,43].

Looking at other studies in which high-intensity exercise is used, we did not come to the same conclusions in terms of biomarkers and locomotor learning [44,45]. Those differences could be explained based on the different design of the study and on other factors such as the intensity, the resting periods or the exercise volumes. It has been observed that the activation of BDNF, lactate or VEGF varies depending on the intensity and duration of the stimuli [15,41]. HIIT is different because the exercise made in intervals allows the management of an anaerobic metabolic stress level that triggers lactate, BDNF and VEGF pathways discussed earlier. Other authors have demonstrated that aerobic exercise can increase the neuroplasticity markers, being a viable option [46]. However, HIIT could be a more efficient alternative because it has a larger work volume of physiological stress in less time [47]. Some authors compared aerobic exercise with HIIT in patients with stroke and found larger increments in BDNF levels and longer maintenance of those BDNF and irisin levels [17]. Exploring this line of research, a meta-analysis regarding BDNF in different exercise modalities found similar results to ours regarding HIIT [48], reassuring the effect of this type of intervention. Expanding on this topic, the study from Boyne et al. (2023) improved walking performance on the 6-MWT with higher preferred walking speed within the HIIT group [27]. The authors correlate this to a higher stepping rate but especially to training speed [49], which is greater in the HIIT group because of the need to reach a higher intensity compared to MICT. Valkenborghs et al. (2019) support that HIIT increases performance in 6-MWT as well [28].

Nonetheless, we found limitations like the scarce number of articles and the variability of the interventions and protocols.

The following limitation of this review is a feature of BDNF related to its gene, whereby there is a polymorphism of a single nucleotide Val66Met that interacts with gender, age and depression, especially in stroke patients [50]. Possible alleles for this can be Val/Val, Val/Met and Met/Met. Recent research points towards the Met/Met allele combination having worse recovery and outcomes when compared to the other alleles [51]. The Met/Met allele can be more common depending on the race, which is observed more in the Asian race while the Caucasians have more representation of the Val/Met allele [51]. However, none of the studies of this review addressed this topic; we suppose that is due to the high complexity of this type of measurement. Other authors point out that the neuronal plasticity of the Met allele relies more on subcortical structures rather than intracortical connections, so it would be important to use rehabilitation techniques that focus more on these aspects and make use of structures like basal ganglia when recovering from stroke [50,52]. Another limitation that we have is the absence of a meta-analysis, the main reason being the lack of homogeneity between studies that would allow a strong meta-analysis, thus making this limitation into a task that could be aborded when the amount of data and standardized studies increase.

Finally, our last limitation is that we limited our search range to works from 2017 onwards, this limitation is due to the fact that HIIT and BNDF were not researched together till that year, thus our choice. However, before that date, there were feasibility and preliminary studies about HIIT and stroke that highlighted its beneficial effects. These studies would not meet the eligibility criteria, but they are the beginning of this line of research, that has taken us to where we are now.

### Future Perspectives

Regarding future studies, a higher number of studies assessing neuroplasticity markers such as BDNF, VEGF or lactate are needed. In addition to evaluating their presence in plasma, it would be necessary to search for these markers in other important areas of the CNS such as the cerebrospinal fluid, where the hypoxia-induced factor (HIF), which is related with directly associated with VEGF expression as well as other neurotrophic factors such as BDNF [53]. It was observed that patients with the highest recovery rate were the ones who had higher levels of HIF [53]. Another study in hypertensive mice observed that the ones who did voluntary exercise fomented the release of fibroblast growth factor receptor 2 in cells located on the third ventricle and cerebrospinal fluid, favoring the neurogenesis on the hypothalamus to help restore the homeostatic functions [54]. Another measurement of interest would be some of the exerkines that are released in response to exercise and influence the brain such as irisin or other neurotrophins such as neurotrophin-3 which promotes neuroplasticity in the dentate gyrus and other metabolites like β-hydroxybutyrate, a ketone body that enhances BDNF expression [6,55].

On the other hand, it would be valuable to look at platelets, because a great amount of BDNF is stored in the alpha granules [41,56]. It has been observed that physical exercise increases BDNF as well as platelet number, assessing if the duration of the increased BDNF correlates with the duration of the elevated number of platelets or if we can prolong the beneficial effects of exercise through platelets.

Although the power of HIIT protocols seems clear, it would be necessary to have studies with early interventions on acute patients, where the destruction of neurons has not advanced and there is a great structural and functional capacity regarding neuroplasticity [57]. Making use of the recovery window which the penumbra area offers from 1 to 12 weeks after the deterioration days since the episode [58].

## 5. Conclusions

HIIT protocols in patients with stroke increase neuroplasticity biomarkers such as BDNF, VEGF and lactate. Although, in order to obtain a more beneficial effect, longer and more intense protocols seem to be the most efficient. More studies are needed regarding neuroplasticity markers and HIIT, as well as looking for other potential exerkines in the chain of reactions produced by exercise that helps in the recovery of stroke.

## Figures and Tables

**Table 1:** Summary of study characteristics and main outcomes.

Authors	Population	Interventions	HIIT	Measures	Outcomes
Nepveu et al., 2017 [23]	n = 22; Time after stroke: >6 months.	Motor task for skill retention (time on target); Rest (Group 1); HIIT (1 session) (Group 2); Motor task for skill retention (time on target).	2 min warm-up (25% peak workload GTX), 3 × 3 min (100% peak workload GTX) with active recovery of 2 min (25% peak workload GTX).	GTX, VO2, HRmax, Pemax, CSE, SICI, ICF, CSP.	HIIT increases skill retention.
Krawcyk et al., 2019 [24]	n = 63; Time after stroke: 3 weeks after episode;	Usual care (medication and lifestyle advice); HIIT (5 days/week, 12 weeks).	3 × 3 min with 2 min of active recovery, 77–93% of the maximum heart rate.	Pro-ADM, Pro-ANP, copeptin, IL-6, TNF, ICAM-1, VCAM-1, VEGF, E-selectin.	Ambiguous response of biomarkers, W of bicycle did not increase.
Boyne et al., 2019 [19]	n = 16; Time after stroke: >6 months.	Treadmill-HIIT; Seated-stepper-HIIT; Seated-stepper-HIIT (revised); Treadmill-MICT (3 sessions); Crossover randomized clinical trial.	Treadmill 20 min of exercise, repeated 30 s bursts at maximum tolerated speed (0% incline). Recovery from 60 to 30 s after 5 min. Seated stepper high-HIIT—same burst and recovery durations as HIIT-treadmill. Bursts at maximum possible cadence against 50% of maximal resistance. Revised HIIT stepper: No resistance, mean intensity target 70–85% HRR. MCT-treadmill walking with aerobic intensity of 45 5% HRR.	Blood lactate, VO2, HRR, CSA CSP, BDNF.	HIIT associated with increases in BDNF, lactate and VO2. BDNF not associated with motor activation threshold or CSP.
Boyne et al., 2020 [20]	n = 16; Time after stroke: >6 months.	Treadmill-HIIT; Seated-stepper-HIIT; Seated-stepper-HIIT (Modified); Treadmill-MICT (3 sessions); Crossover randomized clinical trial.	Same protocol as stated above.	Blood lactate, VEGF, IGF-1, BDNF.	Treadmill HIIT increases VEGF, BDNF and IGF-1. High lactate levels associated to a higher number of neural markers.
Hsu et al., 2021 [25]	n = 23; Time after stroke: >3 months.	MICT; HIIT (36 sessions).	3 min warm-up 30% VO2 peak, 5 × 3 min intervals 80% VO2 peak, 3 min cooldown 30% VO2 peak,	BDNF, neurite growth (%), cerebral tissue. Hb: total hemoglobin, oxyhemoglobin, deoxyhemoglobin.	Increased cerebral blood flow and O2. BDNF increase can result in neural growth.
Abraha et al., 2018 [26]	n = 12; Time after stroke: >6 months.	MICT; HIIT.	5 min warm-up increasing workload till 80%VO2 peak, 5 × 2 min intervals at 80% VO2 peak, 5 × 2 min active recovery at 40% VO2 peak.	VO2 peak, HR, MEP, MEP latency.	HIIT lengthened nerve conduction latency. This effect was intensity-dependent.
Boyne et al., 2023 [27]	n = 55; Time after stroke: >6 months; Gender: M(36) F(19); Type of stroke: I(34) H(21).	MICT; HIIT.	Treadmill 20 min of exercise, repeated 30 s bursts at maximum tolerated speed (0% incline), recovery from 60 to 30 s after 5 min.	Blood lactate, VO2, 6-MWT.	Higher intensity seems better than moderate intensity for improving walking capacity.
Valkenborghs et al., 2019 [28]	n = 20; Time after stroke: >6 months; Gender: M(11) F(9); Type of stroke: I/H (20).	AEX + TST; TST.	4 × 4 min interval, 85%Hrmax, 3 × 3 min active recovery, 70%Hrmax, 5 min light-to-moderate intensity (cooldown).	BDNF, ARAT, WMFT HRmax, VO2max.	Both groups improved their performance in daily activities with HIIT improving retention nearly twice as much as TST alone. However, BDNF decreased in both groups.

**6-MWT**, (six-minute walk test); **AEX**, (aerobic exercise); **ARAT**, (Action Research Arm Test); **CSA**, (corticospinal activation); **CSE**, (corticospinal excitability); **CSP**, (cortical silent period); **GXT**, (graded exercise test); **Hrmax**, (maximal heart rate); **HRR**, (heart rate reserve); **ICF**, (intracortical facilitation); **ICAM-1**, (intercellular adhesion molecule-1); **IL-6**, (interleukin-6); **MICT**, (moderate-intensity continuous training); **MEP**, (motor-evoked potential); **Pemax**, (maximal rate of perceived exertion during the graded exercise test); **Pro-ADM**, (pro-adrenomedullin); **Pro-ANP**, (pro-atrial natriuretic peptide); **SICI**, (short-interval intracortical inhibition); **TNF**, (tumor necrosis factor); **TST,** (task-specific training); **VCAM-1**, (vascular cell adhesion molecule-1); **VEGF**, (vascular endothelial growth factor); **VO2**, (maximal oxygen consumption); **WMFT**, (Wolf Motor Function Test). See Appendix A for further information.

**Table 2:** PEDro Scale’ Scores.

Study	Random Allocation	Concealed Allocation	Groups Similar at Baseline	Participant Blinding	Therapist Blinding	Assessor Blinding	<15% Dropouts	Intention to Treat Analysis	Between Group Difference Reported	Point Estimate and Variability Reported	Total (0 to 10)
Nepveu et al., 2017 [23]	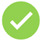	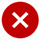	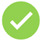	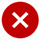	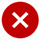	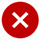	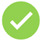	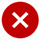	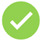	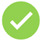	5
Krawcyk et al., 2019 [24]	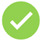	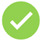	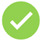	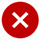	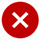	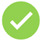	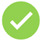	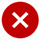	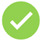	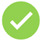	7
Boyne et al., 2019 [19]	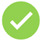	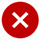	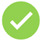	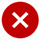	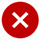	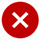	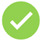	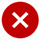	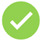	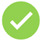	5
Boyne et al., 2020 [20]	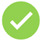	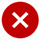	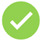	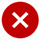	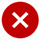	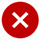	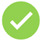	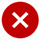	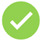	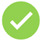	5
Hsu et al., 2021 [25]	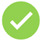	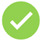	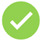	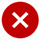	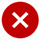	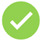	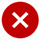	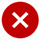	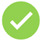	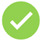	6
Abraha et al., 2018 [26]	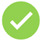	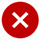	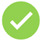	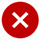	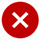	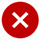	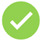	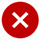	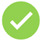	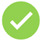	5
Boyne et al., 2023 [27]	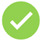	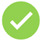	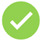	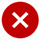	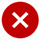	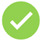	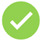	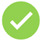	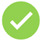	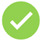	8
Valkenborghs et al., 2019 [28]	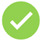	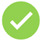	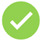	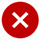	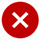	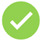	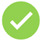	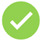	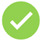	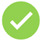	8

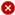
 = Does not meet the criteria 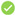 = Does meet the criteria.

## Data Availability

Template data collection forms, data extracted from included studies and other materials used in this review can be accessed through the corresponding author.

## Appendix A. Table Footnote Definitions

6-MWT, (six-minute walk test): The six-minute walk test is a simple cardiopulmonary functional testing modality that can assist in ascertaining the degree of functional impairment and potentially lead to modifications in therapy for some cardiovascular and pulmonary conditions.

AEX, (aerobic exercise): Aerobic exercise is a physical activity that uses the body’s large muscle groups, and is rhythmic and repetitive. It increases heart rate and how much oxygen the body uses.

ARAT, (Action Research Arm Test): A 19-item observational measure used by physical therapists and other health care professionals to assess upper extremity performance (coordination, dexterity and functioning) in stroke recovery, brain injury and multiple sclerosis populations. The ARAT was originally described by Lyle in 1981 as a modified version of the upper extremity function test and was used to examine upper limb functional recovery post damage to the cortex.

CSA, (corticospinal activation): Activation of the corticospinal tract also known as the pyramidal tract. It initiates vascular dilation that delivers oxygen and glucose to the activated region, with increased neural activity, more blood is required to supply metabolic demand.

CSE, (corticospinal excitability): Excitability of the corticospinal tract, based on the expression of plasmalemmal voltage-gated channels. Sensory or synaptic input depolarises (due to generation of net inward current through ligand-gated channels or inhibition of K+ channels by metabotropic pathways) the neuronal plasma membrane above certain thresholds activates voltage-gated ion channels (Na+ channels, K+ channels and to a lesser extent Ca2+ channels). This triggers a regenerative wave of openings and closures of voltage-gated channels along the axon and is recorded in the form of propagating action potentials.

CSP, (cortical silent period): In transcranial magnetic, stimulation is used to assess the activity of the GABAB receptor.

GXT, (graded exercise test): A variety of exercise testing where tests are designed to be increasingly more difficult as they progress. A graded maximal exercise test would ideally progress until the participant reaches a level of maximal exertion, while a graded (multistage) submaximal exercise test would progress to a predetermined point. They are typically administered to determine a participant’s functional aerobic capacity (VO2max), they can also be used to diagnose certain diseases (primarily cardiovascular) when used in conjunction with other diagnostic tools.

Hrmax, (maximal heart rate): The highest heart rate a person can achieve during exercise without experiencing severe problems. It generally decreases with age.

HRR, (heart rate reserve): The difference between your maximum (peak) heart rate and your resting heart rate.

ICF, (intracortical facilitation): Facilitation of an EMG response (motor-evoked potential) by transcranial magnetic stimulation (TMS). ICF is widely assumed to originate from intracortical mechanisms.

ICAM-1, (intercellular adhesion molecule-1): Ig-like cell adhesion molecule expressed by several cell types, including leukocytes and endothelial cells. It can be induced in a cell-specific manner by several cytokines, for example, tumor necrosis factor-alpha, interleukin-1 and interferon-gamma, and inhibited by glucocorticoids. It plays a role in inflammatory processes and on endothelium helps with the migration of (activated) leukocytes to sites of inflammation.

IL-6, (interleukin-6): Member of the pro-inflammatory cytokine family, induces the expression of a variety of proteins responsible for acute inflammation and plays an important role in the proliferation and differentiation of cells in humans.

MICT, (moderate-intensity continuous training): Form of exercise performed at a moderate intensity of Hrmax without rests or stops until the exercise period is completed.

MEPs, (motor-evoked potentials): Potential evoked after a stimulus from transcranial magnetic stimulation.

Pemax, (maximal rate of perceived exertion during the graded exercise test): Maximal rate of perceived exertion during the graded exercise test.

Pro-ADM, (pro-adrenomedullin): More stable precursor peptide to ADM. Once it becomes adrenomedullin, this peptide with a potent vasodilatory effect is regarded as a secretory product of the vascular endothelium.

Pro-ANP, (pro-atrial natriuretic peptide): Precursor of brain natriuretic peptide (BNP) that is used in the identification of heart failure, subarachnoid hemorrhage and carbon monoxide poisoning.

(SICI, short-interval intracortical inhibition): The relative amplitude reduction of motor-evoked potentials (MEPs) by subthreshold conditioning stimuli.

TNF, (tumor necrosis factor): Serum glycoprotein produced by activated macrophages and other mammalian mononuclear leukocytes. It has necrotizing activity against tumor cell lines and increases the ability to reject tumor transplants.

TST, (task-specific training): Task-specific training to improve a specific skill or activity, usually a functional one.

VCAM-1, (vascular cell adhesion molecule-1): Cell adhesion molecule that helps regulate inflammation-associated vascular adhesion and the transendothelial migration of leukocytes, such as macrophages and T cells. Recent evidence suggests that VCAM-1 is closely associated with the progression of various immunological disorders, including rheumatoid arthritis, asthma, transplant rejection and cancer.

VEGF, (vascular endothelial growth factor): Signal protein produced by many cells that stimulates the formation of blood vessels. It is part of the system that restores the oxygen supply to tissues when blood circulation is inadequate such as in hypoxic conditions.

VO2, (maximal oxygen consumption): VO2 max is the maximum rate of oxygen consumption attainable during physical exertion.

WMFT, (Wolf motor function test): It is a quantitative measure of upper extremity motor ability through timed and functional tasks. It is widely used for stroke and brain injury recovery assessment and has reliability and validity data.

## Appendix B. Search Strategy

PubMed: Records identified—1007
Strategy used (using MESH):
(((((“chronic stroke” OR “Stroke”[Mesh]) AND “High-Intensity Interval Training”[Mesh] OR HIIT)
AND “Neuronal Plasticity”[Mesh]) OR “Nerve Growth Factors”[Mesh]) OR “Nerve Growth
Factor”[Mesh]) OR “Brain-Derived Neurotrophic Factor”[Mesh]) OR “Functional Magnetic
Resonance Imaging” OR “Functional MRI”)
Filters used: RCT, Last 5 years, Human.
LILACS: Records identified—3
Strategy used:
stroke AND (“high-intensity interval training” OR HIIT) AND “neuronal plasticity”
Filters used: RCT, Last 5 years,
ProQuest: Records identified—156
Strategy used:
(“chronic stroke” OR stroke) AND (“high-intensity interval training” OR HIIT) AND (“neuronal
plasticity” OR “Nerve growth factors” OR “Nerve growth factor” OR “Brain-derived
neurotrophic factor” OR “Functinoal magnetic resonance imaging” OR “Functional MRI”)
Filters used: Scientific magazine, Last 5 years, articles.
PEDro: Records identified—11
Strategy Used:
“Stroke” AND “High-intensity interval training”
Filters used: Articles from 01/01/2017 and RCT
Web of Science (WOS): Records identified—6
Strategy used:
(“chronic stroke” OR stroke) AND (“high-intensity interval training” OR HIIT) AND (“neuronal
plasticity” OR “Nerve growth factors” OR “Nerve growth factor” OR “Brain-derived
neurotrophic factor” OR “Functinoal magnetic resonance imaging” OR “Functional MRI”)
Filters used: Years 2017–2022, articles.
